# Wood Anatomy of Douglas-Fir in Eastern Arizona and Its Relationship With Pacific Basin Climate

**DOI:** 10.3389/fpls.2021.702442

**Published:** 2021-09-01

**Authors:** Daniel Balanzategui, Henry Nordhauß, Ingo Heinrich, Franco Biondi, Nicholas Miley, Alexander G. Hurley, Emanuele Ziaco

**Affiliations:** ^1^GFZ German Research Centre for Geosciences, Potsdam, Germany; ^2^Institute of Geography, Humboldt-University, Berlin, Germany; ^3^Department of Natural Sciences, DAI German Archaeological Institute, Berlin, Germany; ^4^DendroLab, Department of Natural Resources & Environmental Science, University of Nevada, Reno, NV, United States; ^5^Department of Ecology and Genetics, Plant Ecology and Evolution, University of Uppsala, Uppsala, Sweden

**Keywords:** quantitative wood anatomy, *Pseudotsuga menziesii*, El Niño/Southern Oscillation, Pacific Decadal Oscillation, paleoclimate, western United States

## Abstract

Dendroclimatic reconstructions, which are a well-known tool for extending records of climatic variability, have recently been expanded by using wood anatomical parameters. However, the relationships between wood cellular structures and large-scale climatic patterns, such as El Niño-Southern Oscillation (ENSO) and the Pacific Decadal Oscillation (PDO), are still not completely understood, hindering the potential for wood anatomy as a paleoclimatic proxy. To better understand the teleconnection between regional and local climate processes in the western United States, our main objective was to assess the value of these emerging tree-ring parameters for reconstructing climate dynamics. Using Confocal Laser Scanning Microscopy, we measured cell lumen diameter and cell wall thickness (CWT) for the period 1966 to 2015 in five Douglas-firs [*Pseudotsuga menziesii* (Mirb.) Franco] from two sites in eastern Arizona (United States). Dendroclimatic analysis was performed using chronologies developed for 10 equally distributed sectors of the ring and daily climatic records to identify the strongest climatic signal for each sector. We found that lumen diameter in the first ring sector was sensitive to previous fall–winter temperature (September 25^th^ to January 23^rd^), while a precipitation signal (October 27^th^ to February 13^th^) persisted for the entire first half of the ring. The lack of synchronous patterns between trees for CWT prevented conducting meaningful climate-response analysis for that anatomical parameter. Time series of lumen diameter showed an anti-phase relationship with the Southern Oscillation Index (a proxy for ENSO) at 10 to 14year periodicity and particularly in 1980–2005, suggesting that chronologies of wood anatomical parameters respond to multidecadal variability of regional climatic modes. Our findings demonstrate the potential of cell structural characteristics of southwestern United States conifers for reconstructing past climatic variability, while also improving our understanding of how large-scale ocean–atmosphere interactions impact local hydroclimatic patterns.

## Introduction

The climate of the North American West is closely connected with ocean–atmosphere variability in the Pacific Basin, which is often defined through indices, such as the El Niño-Southern Oscillation (ENSO) index (Barnett et al., 1991) and the Pacific Decadal Oscillation (PDO) index (Mantua and Hare, 2002). Both climatic modes play a fundamental role in determining interannual variability of ecohydrological conditions, affecting water resources, particularly in the moisture-limited environments of the western United States (Corringham and Cayan, 2019; White et al., 2019), and long-term vegetation dynamics (Viglizzo et al., 2015). Over the western United States, the southward shift of convective air masses that occurs over the ocean during an El Niño (La Niña) event often results in above (below) average winter rainfall over the southwest (Sheppard et al., 2002). Similarly, warm (cold) PDO phases are positively (negatively) correlated with winter precipitation along the coast of southern California, Arizona, and New Mexico (Mantua et al., 1997). Recently, model predictions based on large-scale atmospheric circulation patterns have been shown to unsatisfactorily reproduce observed precipitation dynamics (Jong et al., 2018). This is because linking ENSO- and PDO-driven regional climatic conditions with local hydroclimatic variability requires properly accounting for additional site-specific factors. For example, wind direction and local topography can cause abnormally high rainfalls to occur, contrary to expectation, during La Niña events in the southwest United States (Feldl and Roe, 2010).

Since instrumental records of ENSO and PDO begin in the late 19^th^ century, the range of local climate variability that can be expected during different modes of ENSO and PDO can be better estimated by extending instrumental observations using proxy records, such as climate-sensitive tree-ring chronologies (Biondi et al., 2001; Stahle et al., 2009; Woodhouse et al., 2013; Ziaco et al., 2020). The inclusion of cellular-level wood anatomical traits in dendroclimatological studies has the potential to improve the spatiotemporal coverage of hydroclimatic reconstructions (González-Cásares et al., 2019; Seftigen et al., 2020). Quantitative wood anatomy (QWA) in dendroclimatology, namely, the response between woody cell structures and climatic variability (von Arx et al. 2016), has been applied to angiosperm (García-González and Eckstein, 2003; Tardif and Conciatori, 2006; Fonti and García-Gonzalez, 2008; Campelo et al., 2010) and conifer species (Wang et al., 2002; Panyushkina et al., 2003; DeSoto et al., 2011; Liang et al., 2013b), but cellular-level dendroclimatology in the western United States is still relatively new (Ziaco et al., 2016; Edwards et al., 2020). Intra-annual variability of xylem structures depends on resource allocation and trade-offs between plant growth and reproduction that are driven by local hydroclimatic variability (Lauder et al., 2019) at short (sub-seasonal) time scales (Bouriaud et al., 2005). Hence, annually resolved time series of cellular parameters often present stronger or different relationships with seasonal climate compared to their ring-width counterpart (Castagneri et al., 2017), offering studies with limited wood material the potential to overcome some of the restrictions of tree-ring-based paleoclimatic reconstructions, such as the minimum sample size (Pearl et al., 2020). For instance, multi-centennial reconstructions of summer (Panyushkina et al., 2003) and winter temperature (Pritzkow et al., 2016) have been produced using wood anatomical parameters extracted from a limited number of samples otherwise deemed insufficient for standard ring-width analysis. Furthermore, the partitioning of tree rings into sub-sections (herein referred to as “sectors”) is a rapidly growing technique with the potential to improve the temporal resolution of the climatic signal encoded in intra-annual xylem cellular structures (Carrer et al., 2017). Tree-ring partitioning, either in equal parts (Castagneri et al., 2017) or between earlywood and latewood (Fonti and García-González, 2004; Torbenson et al., 2016), better characterizes intra-seasonal changes in wood anatomical features along the tree ring and is expected to enhance the temporal resolution of climate-anatomy relationships while also clarifying and expanding the amount and quality of environmental information obtainable from trees (Ziaco and Liang, 2019).

Douglas-fir [*Pseudotsuga menziesii* (Mirb.) Franco] is one of the most widely used species for dendroclimatic research in the western United States. It is widely distributed along the coast of the American northwest and western Canada, and from Mexico to the Rocky Mountains (Li and Adams, 1989), it can reach ages greater than 1,000years (Contributors of the International Tree-Ring Data Bank, 2020) and its ring-width chronologies are typically sensitive to climate. It is therefore an ideal target for testing the potential of cellular-level dendroclimatology. The main goal of this explorative study was to assess the utility of wood anatomy of *P. menziesii* for dendroclimatic reconstructions with particular focus on Pacific Basin climate, and specifically, 1) to test the temporal and spatial climate relationships of intra-annual xylem anatomical parameters in *P. menziesii* and 2) to evaluate the potential contribution of these proxies for bridging the gap between large-scale atmospheric circulation modes (such as ENSO and PDO) and local hydroclimatic variability. To achieve these goals, we developed ring-sector chronologies and assessed the strength of the climatic signal encoded in their wood anatomical structures.

## Materials and Methods

### Study Area

**Figure 1:**
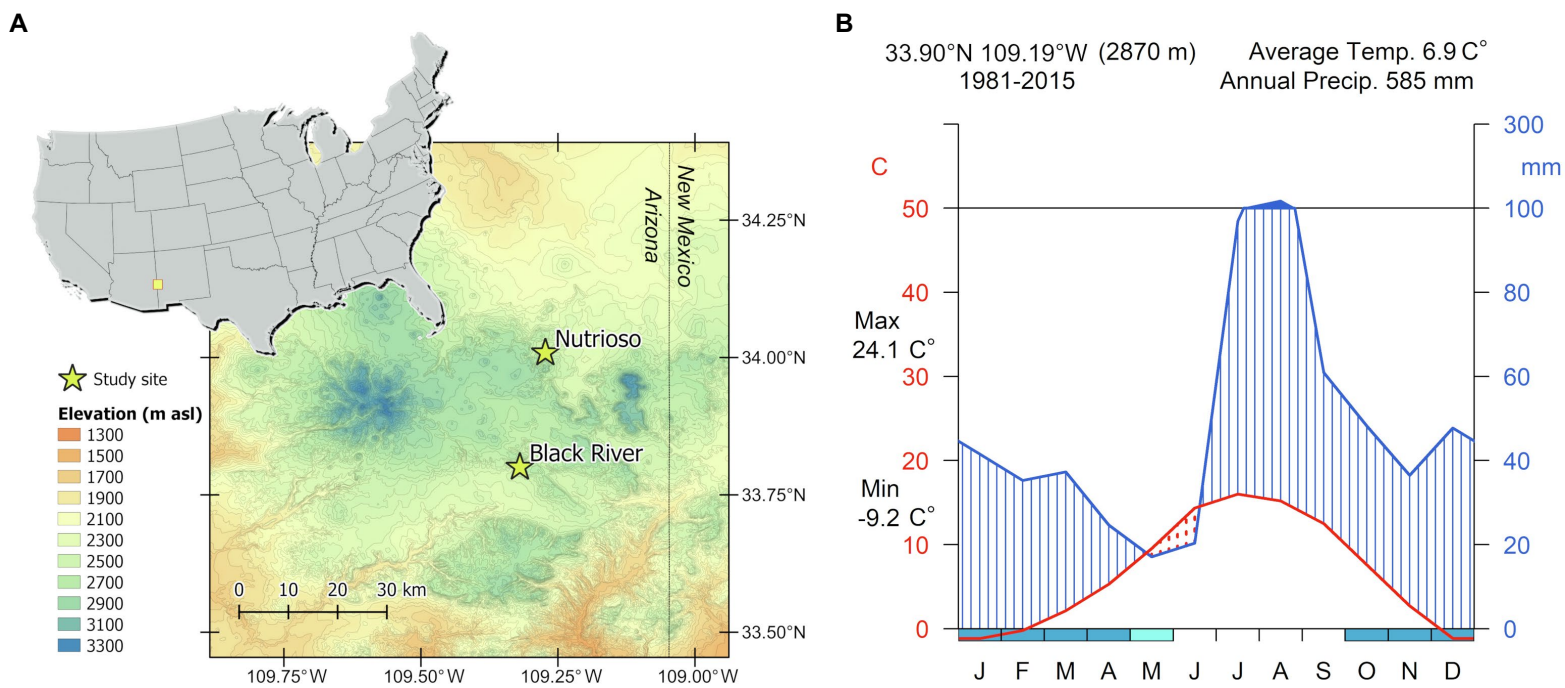
**(A)** Geographical location of the two study sites. **(B)** Walter-Lieth climatic diagram for the period 1981–2015 based on PRISM data.

The study site, located in the White Mountains of eastern Arizona on the southern edge of the Colorado Plateau (Figure 1A), was preselected based on a preliminary analysis of the site characteristics performed with the web-based tool Dendrobox (Zang, 2015). Despite falling within the core region of the North American Monsoon (NAM), where precipitation is mostly concentrated in the summer months (Griffin et al., 2013), mountain areas in this part of the southwestern United States are also sensitive to cool-season conditions linked to ENSO, and the Dendrobox tool allowed us to search the International Tree-Ring Data Bank (Grissino-Mayer and Fritts, 1997) for existing sites with ring-width chronologies sensitive to winter precipitation. Tree samples were collected in forest stands dominated by *P. menziesii* near the village of Alpine, Arizona. Trees were located at altitudes between 2,500 and 2,900ma.s.l. on south- to southeast-facing slopes with 25–52% steepness.

The topographically complex landscape of the White Mountains is influenced by multiple climatic patterns linked to the Pacific Ocean, including the Gulf of California, and the Atlantic Ocean, including the Gulf of Mexico (Jana et al., 2018). The precipitation regime reflects the typical bimodal pattern found in the southwestern United States (Figure 1B), with a peak in late winter derived from large-scale frontal storms originating over the eastern Pacific, followed by summer rainfall carried inland by the NAM (Sheppard et al., 2002). Daily time series of maximum temperature (Tmax) and total precipitation for the years 1981–2015 were obtained from the PRISM (Parameter-elevation Relationships on Independent Slopes Model) 4km dataset (Daly et al., 2008) at the grid point 33.90° N 109.19°W. The climate during this period was characterized by a mean annual temperature of 6.9±3.2°C, with January and July being the coldest (−1.2±3.8°C) and warmest months (16.0±1.8°C), respectively. Total annual precipitation was 585±113mm, with a substantial amount falling from July to August (37% of the annual total), and snowpack lasting on average from November to March (Figure 1B).

### Tree-Ring Chronologies and Anatomical Measurements

In April 2016, a total of 31 increment cores were collected from two sites (Nutrioso and Black River; Figure 1A) from healthy *P. menziesii* trees with diameters at breast height (~1.3m from the ground) ranging from 45cm to 93cm, and an average height of 35m. Increment cores were taken with a 4mm increment borer in a direction parallel to topographic contours to avoid compression wood produced by gymnosperms on the downhill side of the stem (Speer, 2010). Increment cores were mounted with wood tracheids oriented vertically, sanded with progressively finer sandpaper, and finally hand polished until individual cells were visible under 10-50x magnification. Individual series were cross-dated and measured on a Velmex sliding-scale micrometer table interfaced with a video camera with the Measure J2X software (VoorTech Consulting), and finally averaged to develop a ring-width chronology (Supplementary Figure S1a). Trees from Nutrioso were on average 118±35years old with minimum and maximum ages of 92 and 250years, respectively, while trees sampled at Black River were 142±35years old on average and ranging from 83 to 174years. The mean ring width was 1.47±0.37mm at Nutrioso and 1.62±0.41mm at Black River. Raw ring-width series were standardized using a 50-year smoothing spline with 0.5 frequency cutoff and pre-whitened to remove first-order autocorrelation. As a preliminary step, we tested climate-growth relationships for the period 1950–2015 using monthly data of Tmax and precipitation. Response functions showed that *P. menziesii* radial growth at these sites is positively influenced by previous December to current January precipitation, while also negatively affected by July temperature (Supplementary Figure S1b).

**Figure 2:**
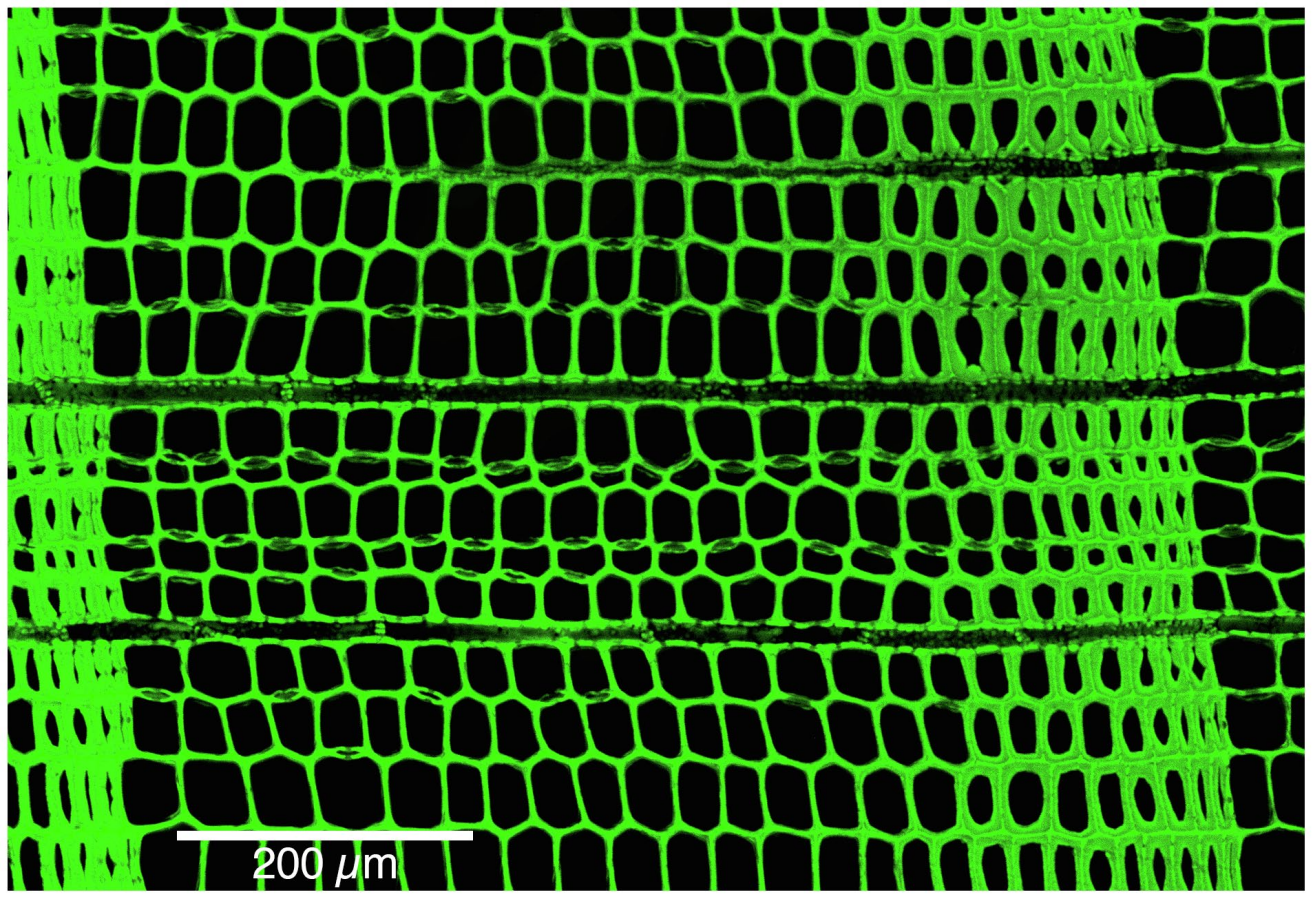
Microscopic composite (merged) image of *Pseudotsuga menziesii* at 300x magnification with brightness correction.

The wood anatomical analysis was conducted on a subset of five trees (three from Nutrioso and two from Black River) by selecting cores with high-quality intra-annual xylem structures, and absence of surface cracks or other alteration of the woody tissue. For the extraction of resinous compounds, samples were separated from their mounts, divided into 5-cm long pieces, and submerged in glass vessels filled with 99.9% isopropanol for 48h in a 70° C ultrasonic water bath operating at a frequency of 37kHz. Samples were then air dried, remounted, and stabilized using non-Newtonian fluid to avoid cell wall breakage during the cutting procedure (Schneider and Gartner, 2013). Micro sections (~20μm) were cut from the transversal surface with an advanced core-microtome (Gartner and Nievergelt, 2010). Thin sections were then stained with 1% safranin diluted in distilled water, dehydrated with alcohol, and temporarily fixed in glycerol between two glass microscope slides (Gärtner and Schweingruber, 2013). Sequential micro-images were taken at 100x magnification following the procedures and settings described by Liang et al. (2013a) using an Olympus FluoView FV300 Confocal Laser Scanning Microscope (CLSM) that displays cell wall (green) and lumen (black) in strong contrast (Figure 2). Large, single frame images derived from composite CLSM micrographs (25 to 30 images) were processed with the quantitative wood anatomical program, ROXAS 3.0.1 (von Arx and Carrer, 2014 and supporting software Image-Pro Plus v6.1 Media Cybernetics, Silver Spring, MA, United States), to identify, date, edit, and measure all cell structures. Although CLSM allows to acquire images directly on the wood surface, in our set of samples we found the wood properties of the inner part of the tree, where the rings are darker, to be more suitable for direct surface scanning, whereas the lighter sap wood rings were not, requiring additional steps of staining and thin sectioning to allow CLSM imaging to enhance the contrast between cell structures. As in this study we focused on the outermost, light-colored sapwood rings, we preferred to use thin sections, which provided better results although more time-consuming.

From the QWA data produced by ROXAS, our analysis focused on the intra- and inter-annual variability in radial diameter of cell lumen (LD) and tangential cell wall thickness (CWT). The QWA specific search algorithms of the R package, RAPTOR 1.0.1 (Row and Position Tracheid Organizer in R), were used to locate cell rows and assign each cell (tracheid) and corresponding QWA measurements to the correct position within its respective radial file (Peters et al., 2018). Depending on the quality of the scanned material and the number of resin ducts within each tree ring, between 10 to 25 cell rows were identified, exceeding the number usually recommended (Seo et al., 2014). While the distance across each ring was mostly homogenous the number of cells within each cell row showed some variation, therefore standardized profiles (tracheidograms) for each cell file were produced to better illustrate intra-seasonal and year-to-year site-wide growth patterns along the tree ring (Vaganov, 1990; Ziaco, 2020). Tracheidograms for each radial file, year, and tree were divided into 10 equal sized sectors (from I to X). While tree-ring partitioning is generally performed by visually dividing the ring into 10 equally spaced portions based on ring width, in our approach the location of each sector boundary was determined for each analyzed row in relation to the total number of cells found in that row – an analytical step made possible with the inclusion of the RAPTOR algorithms. With this approach, the cells of each radial file could be precisely assigned to their respective sectors. The simple average of LD or CWT measurements for all cells falling within a specific sector within the row and ring was then calculated to produce 10 sectorial chronologies for each anatomical parameter. Finally, the 10 sector chronologies of each tree were normalized (zero mean and unit standard deviation) across the common period 1966–2015, and the average taken across all trees to obtain sector chronologies revealing site-wide, intra-seasonal anatomical variability. Due to lack of a long-term trend, which usually occurs in ring-width series because of inherent non-climatic trends interfering with the climatic signal, no detrending procedure was applied (Carrer et al., 2017). Chronology descriptive statistics commonly employed in tree-ring studies were used to gage the quality of the common variance (i.e., climatic signal) shared among trees (Hughes et al., 2011). These included mean inter-series correlation (rbar), expressed population signal (EPS), and signal-to-noise ratio (SNR). Descriptive statistics were calculated using the R package *dplR* (Bunn et al., 2014).

### Dendroclimatic Analysis

Each sector chronology was compared with daily Tmax and total precipitation (PPT) from 1981 to 2015 using the Climate Impact on Tree Growth (CLIMTREG) program (Beck et al., 2013). CLIMTREG initially calculates correlations between climate and anatomical parameters for a 21-day-long period, then iteratively adds one day to the correlation window until a maximum interval length of 121days is reached. The moving correlation procedure starts on July first of the previous year and stops on October 31^st^ of the current year, resulting in 42,218 correlations calculated per run. Hence, the starting and ending date of climate-anatomy correlations could be identified with daily resolution, summarized for each climatic parameter (temperature and precipitation), and visualized for each tree-ring sector. Since our interest was in evaluating the potential of wood anatomy for dendroclimatic reconstructions, whenever multiple significant correlations with climate emerged we identified and retained only the period most strongly correlated with a given parameter and used only that respective window for further analyses and visualization.

The sectors that showed acceptable chronology statistics (Wigley et al., 1984) and relationships with daily climate parameters were chosen to assess spatial field correlations. The spatial signature of the growth response to climate was investigated using the web-based KNMI Climate Explorer tool^1^ (Trouet and Van Oldenborgh, 2013) to generate correlation fields over the United States and Mexico, as well as the Pacific, Gulf of Mexico, and Atlantic regions. Because daily data are not available for spatial field correlation in KNMI Climate Explorer, this analysis was done using monthly climate data. Temperature and precipitation were taken from the CRU TS4.03 dataset with 50km x 50-km grid cells (Mitchell and Jones, 2005). Sector chronologies were also correlated against 1-month Standardized Precipitation and Evaporation Index (SPEI-1; CSIC 2.6; Beguería et al., 2014) and sea surface temperature (SST; NOAA/NCEP Reynolds OI.v2; Reynolds et al., 2002) to investigate the influence of interdecadal or decadal climatic oscillations patterns derived from ENSO and PDO.

Finally, the relationship between wood anatomy in *P. menziesii* and supra-regional climatic modes was quantified by applying a cross-wavelet transform (Grinsted et al., 2004) to annually resolved time series of anatomical parameters for selected sectors against the Southern Oscillation Index (SOI; Ropelewski and Jones, 1987) and PDO (Mantua et al., 1997) indices over the period 1966–2015. The SOI is a measure of fluctuations in air pressure occurring between the western and eastern tropical Pacific and is one of the most commonly used proxy indicators to describe anomalies in ENSO behavior (Ropelewski and Jones, 1987). Negative (positive) phases of the SOI are associated with El Niño (La Niña) conditions that often result in above (below) average precipitation in the southwestern United States. Cross-wavelet analysis highlights common time frequency phase variations, phase cyclicity, and links between different bio-geophysical proxies (Wang et al., 2011; Land et al., 2020). Explanatory monthly variables of SOI and PDO series were pre-determined based on the outcomes of the dendroclimatic calibration. Cross-wavelet analysis was performed using the package *WaveletComp* (Roesch and Schmidbauer, 2018) for the R statistical environment (R Core Team, 2021).

## Results

### Cell Structure and Sector Chronologies

**Figure 3:**
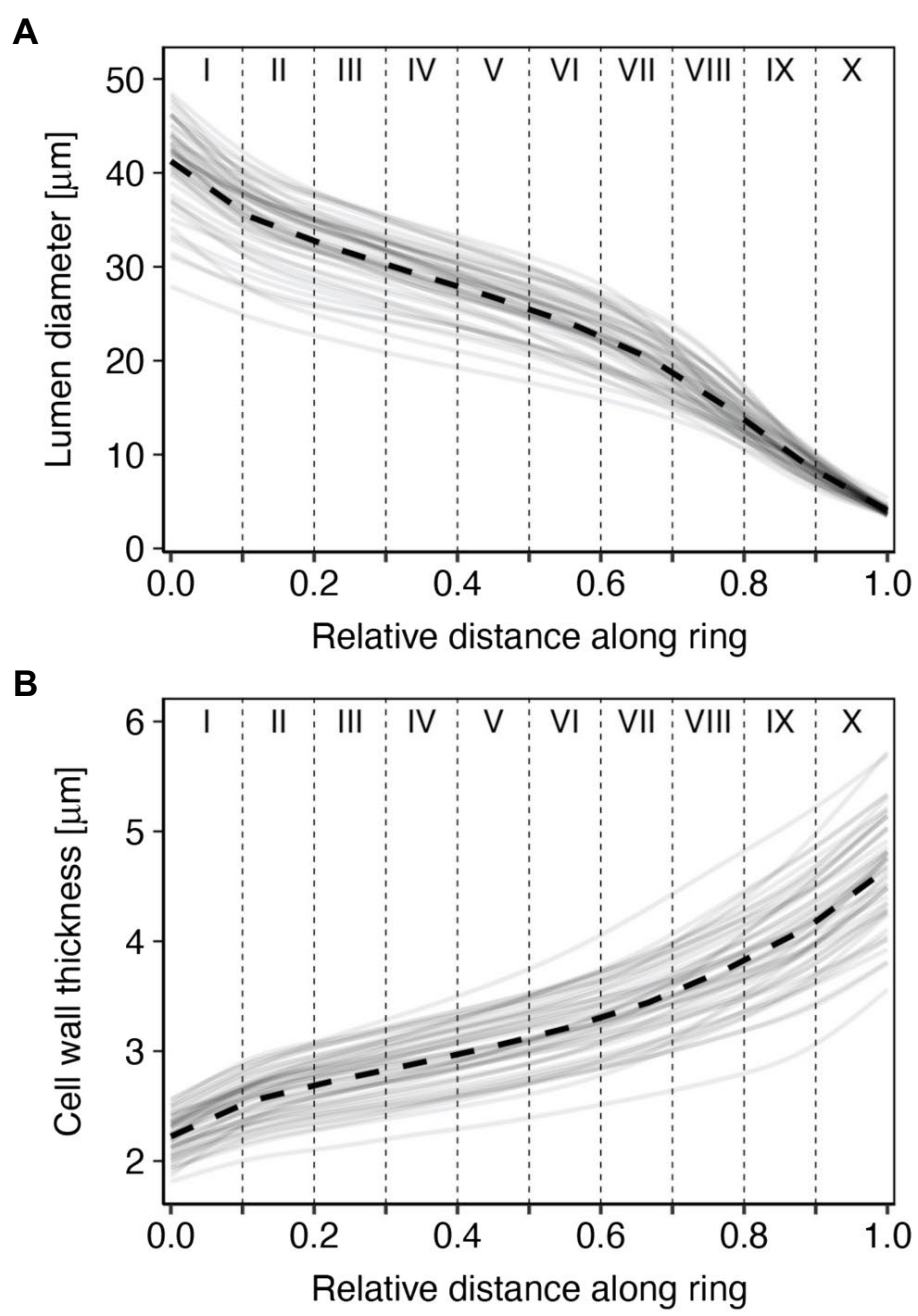
Annual (gray solid lines) and mean (black dashed lines) standardized tracheidograms of **(A)** lumen diameter and **(B)** cell wall thickness (CWT) for the five sampled trees at Nutrioso and Black River for the years 1966–2015. Roman numerals indicate tree-ring sectors, from the beginning (sector I) to the end of the ring (sector X).

**Figure 4:**
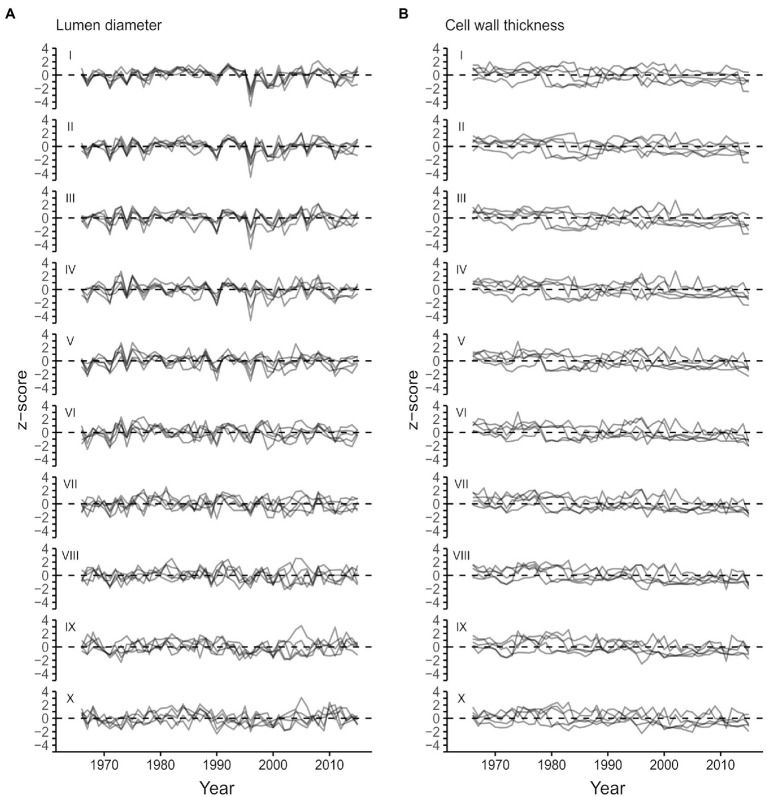
Standardized sector chronologies for the period 1966–-2015 for lumen radial diameter **(A)** and CWT **(B)** moving from the beginning (sector I) to the end (sector X) of the ring.

From the start to the end of the tree ring, lumen diameter (LD) decreased from 41.3±4.7μm in the first part of the ring (sector I) to 4.0±0.4μm in the last part (sector X; Figure 3A; Table 1). CWT instead increased from about 2.2±0.2μm in the first sector (I) to 4.7±0.5μm in the last sector (X; Figure 3B; Table 1). Site-averaged sector chronologies of LD had negligible first-order autocorrelation ranging from 0.07 to 0.32, whereas first-order autocorrelation was higher for CWT chronologies, ranging between 0.62 and 0.73 (Table 1). Inter-annual variability of LD between the five trees generally showed stronger synchronization in the earlier sectors compared to the later ones (Table 1, Figure 4A). These common growth patterns from the first to the fifth sector were shown by rbar values from 0.438 to 0.658, EPS from 0.796 to 0.906, and SNR from 3.496 to 6.368. On the other hand, CWT series were noisy along the entire ring (rbar from 0.004 to 0.166, EPS from 0.026 to 0.501, and SNR from 0.012 to 1.314) and generally lacked common variability between trees (Table 1, Figure 4B). For this reason, CWT chronologies were excluded from further analysis.

**Table 1:** Summary statistics of cell lumen diameter and cell wall thickness chronologies.

	Lumen diameter (LD)					Cell wall thickness (CWT)				
Sector	Mean±SD	AC1	rbar	EPS	SNR	Mean±SD	AC1	rbar	EPS	SNR
I	41.3 ± 4.7	0.07	0.658	0.906	6.368	2.2 ± 0.2	0.73	0.004	0.026	0.012
II	35.3 ± 3.8	0.04	0.623	0.893	5.755	2.5 ± 0.2	0.76	0.108	0.379	0.634
III	32.1 ± 3.5	0.05	0.504	0.836	4.193	2.7 ± 0.2	0.72	0.166	0.501	1.314
IV	29.5 ± 3.3	0.10	0.447	0.802	4.051	2.9 ± 0.3	0.67	0.137	0.444	0.837
V	26.9 ± 3.1	0.14	0.438	0.796	3.496	3.0 ± 0.3	0.65	0.115	0.397	0.962
VI	24.0 ± 3.0	0.16	0.243	0.616	1.697	3.2 ± 0.3	0.62	0.089	0.332	0.829
VII	20.3 ± 2.7	0.16	0.119	0.402	0.632	3.4 ± 0.3	0.62	0.066	0.263	0.698
VIII	15.0 ± 2.0	0.17	0.093	0.338	0.531	3.8 ± 0.4	0.63	0.045	0.194	0.508
IX	8.8 ± 1.0	0.32	0.108	0.375	0.602	4.1 ± 0.4	0.62	0.015	0.076	0.158
X	4.0 ± 0.4	0.22	0.159	0.486	0.893	4.7 ± 0.5	0.62	0.011	0.062	0.172

Mean and standard deviation (SD = ± one standard deviation, both shown in μm), one-year lag autocorrelation (AC1), series inter-correlation (rbar), expressed population signal (EPS), and signal-to-noise ratio (SNR) were calculated for the period 1966 to 2015, moving from the start of the earlywood (sector I) to the end of the latewood (sector X).

### Correlations Between Anatomical Chronologies and Daily Climate

**Figure 5:**
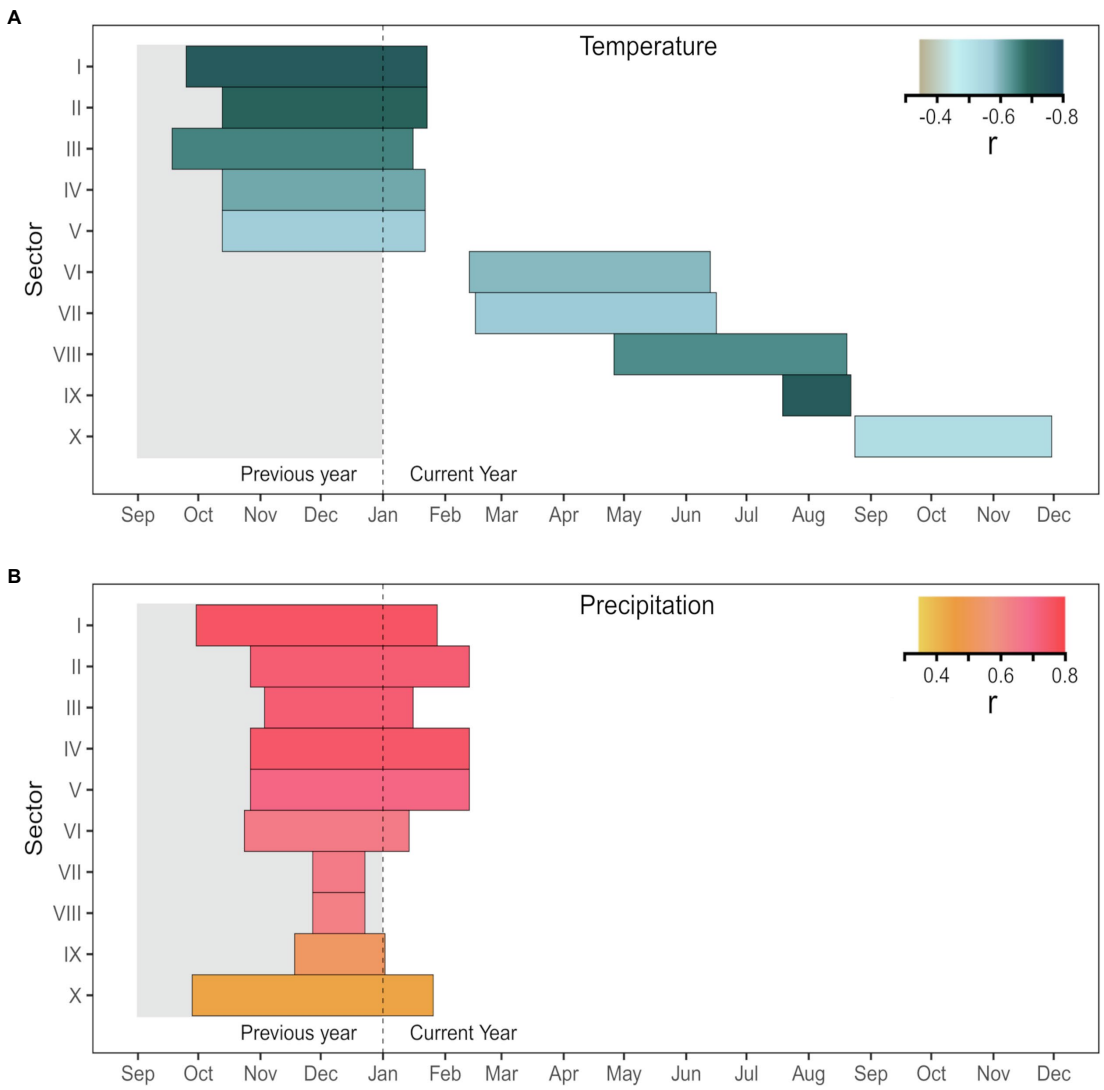
Correlations of sector chronologies of lumen diameter with daily **(A)** maximum temperature (Tmax) and **(B)** precipitation for the period 1981 to 2015. The horizontal length of each bar corresponds to the strongest optimal period identified by the moving window correlation analysis. All correlations, whose value is given by the color, are significant at *p*<0.05.

**Figure 6:**
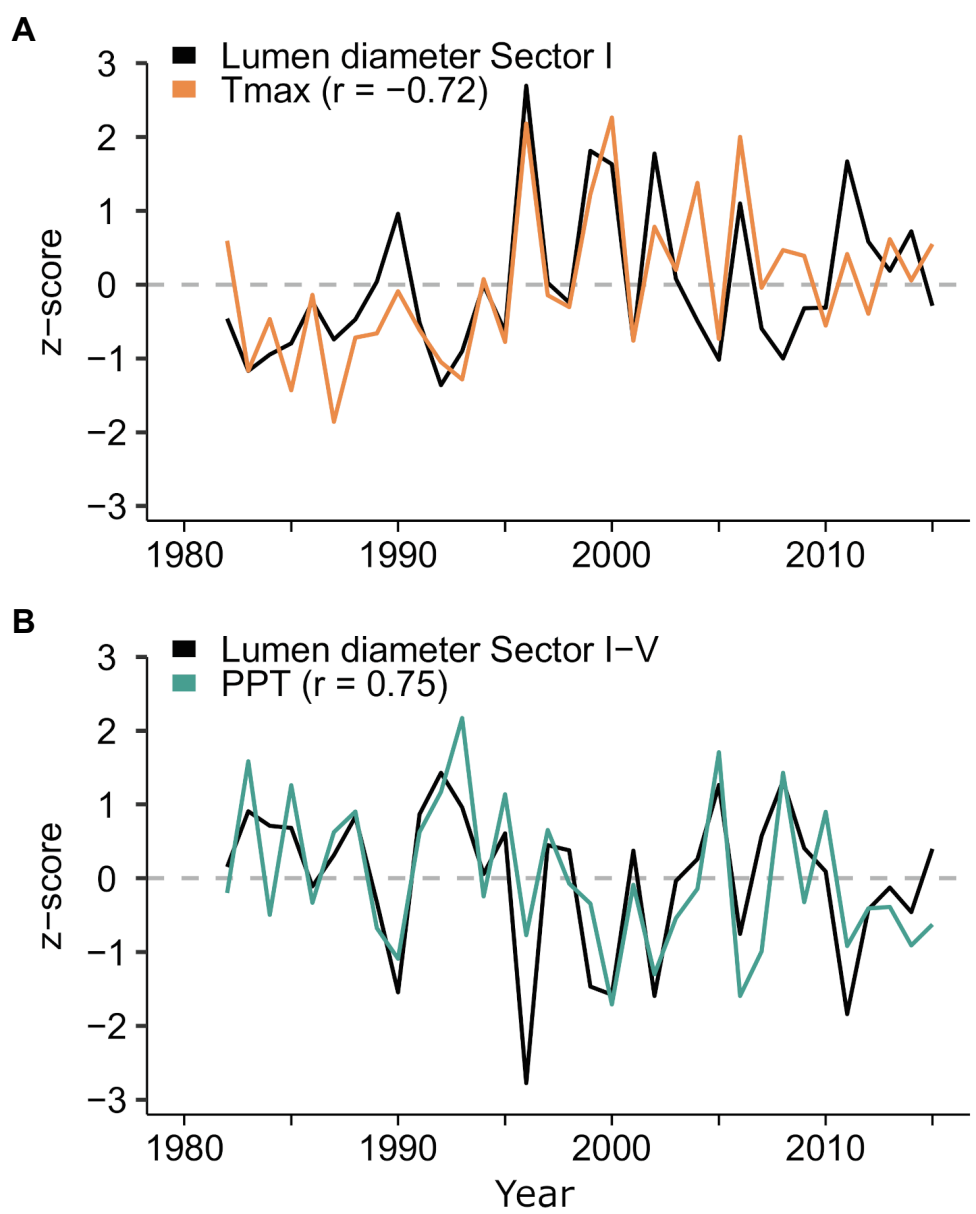
Normalized time series of radial lumen diameter chronologies for sector I and for the average between sectors I-V plotted against **(A)** temperature and **(B)** precipitation. Climate data are the average daily Tmax from September 25^th^ to January 23^rd^, and the sum of daily precipitation from previous October 27^th^ to current February 13^th^. The sector I chronology of lumen diameter is shown with opposite sign for easier comparison with the temperature time series.

Several significant (*p<0.05*) climate-anatomy relationships were found between all LD sectors and daily Tmax and precipitation. The strongest correlations for the first five sectors were with previous mid-September to current mid-February, and generally decreased in strength toward the transition zone between earlywood and latewood cells. Temperature was negatively correlated with LD, with Pearson’s correlation coefficient *r* between −0.56 and−0.72 (Figure 5A), whereas precipitation had a positive correlation, ranging between 0.7 and 0.75 (Figure 5B). For sectors six to 10, the relationship between LD and temperature remained negative, but the temporal window of maximum correlation progressively shifted toward the spring and summer of the current growing season as sector number increased. In the second half of the tree ring, LD maintained a positive linkage with previous-September to current-February precipitation. Maximum dendroclimatic correlations in the latter half of the tree ring were usually weaker than those in the first half. However, sector IX, despite showing poor signal quality (rbar=0.108, EPS=0.375, SNR=0.602), had a strong negative correlation with mid-July to mid-August temperature (r=−0.72). Overall, the first sector (I) carried the strongest signal, responding to Tmax from previous September 25^th^ to current January 23^rd^ (Supplementary Figure S2). The average of sectors I-V showed high correlation (r=0.75) with rainfall between previous October 27^th^ and current February 13^th^ (Figure 6 and Supplementary Figure S3). Based on these results, sector I and the mean of sectors I-V were chosen for further calibration analysis.

### Spatial Field Correlations and Cross-Wavelet Analysis

**Figure 7:**
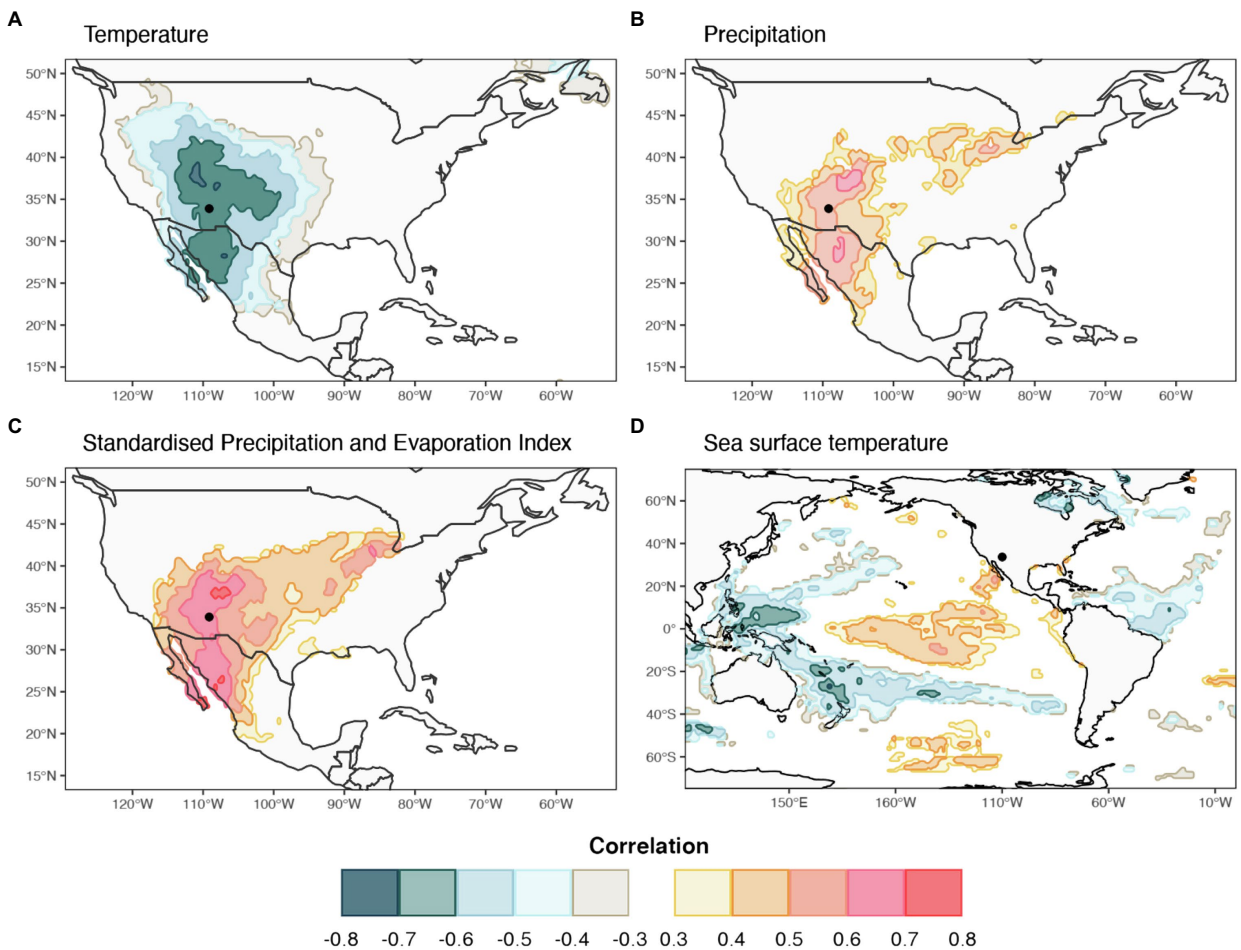
Spatial field correlation maps between selected radial lumen diameter sector chronologies and climate parameters for the period 1981–2015. Sector I chronology was correlated against **(A)** mean October to January temperature (**C**) October to January one-month Standardized Precipitation and Evaporation Index, and **(D)** October to January sea surface temperature. Mean chronology for sectors I-V was correlated against **(B)** November to January precipitation. The location of the study site is identified by a black dot. Colored areas show correlations significant at p<0.05.

Spatial field correlations were performed between lumen diameter chronologies for sector I and for the average of sectors I-V against October–January average Tmax, SPEI-1, SST, and November–January total PPT. The negative correlation between Tmax and LD in sector I was spatially consistent and significant throughout the entire southwestern United States and northern Mexico, with correlations ranging from r=−0.34 to r=−0.71 (Figure 7A). A similar geographical pattern, but positive and with an extension reaching into the American mid-west, was found between sector I-V LD and PPT during previous November to current January (Figure 7B) with correlations ranging between r=0.34 and r=0.70. Spatial correlations between sector I-V LD and SPEI-1 were almost identical in terms of geographical distribution to those with PPT (*r* from 0.34 to 0.75) but were slightly higher and more homogeneously distributed over the southwestern United States, especially Arizona, New Mexico, and southern Colorado (Figure 7C). The sector I-V LD chronology showed widespread significant relationships with October to January SSTs in the Pacific Ocean, resembling the typical pattern of SST anomalies associated with ENSO events (Figure 7D). Correlations between LD in the first half of the ring and SST were positive in the eastern Pacific (r from 0.34 to 0.57), and negative in the western and southern Pacific (r from −0.34 to −0.69).

**Figure 8:**
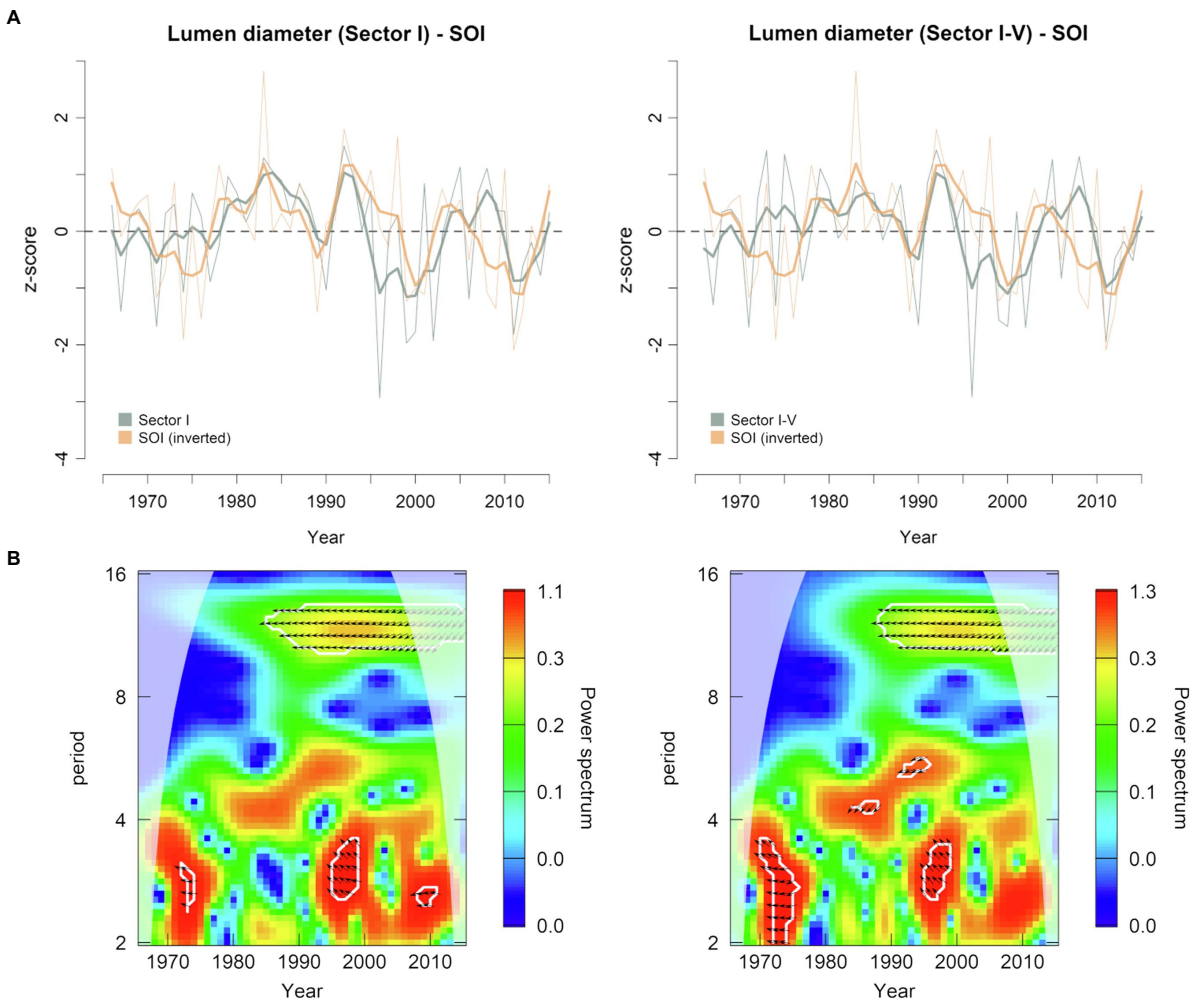
**(A)** Normalized time series of earlywood lumen diameter (sectors I and I-V) plotted against previous October-current January Southern Oscillation Index (SOI) for the period 1966 to 2015 (bold lines are 5-year cubic smoothing splines). The sign of SOI has been inverted for easier interpretation. **(B)** Cross-wavelet transform of lumen diameter (sectors I and I-V) and SOI index. White contours represent 95% significance level. The relative phase relationship is shown as arrows (in-phase pointing right, anti-phase pointing left, and SOI leading lumen diameter by 90° pointing down). Results falling outside the cone of influence (white shaded area) might be distorted by edge effect.

The October–January SOI index and LD were correlated between 1966 and 2015 (Figure 8A). This relationship was higher during the period 1980–2005, when the cross-wavelet power showed a significant anti-phase relationship on the 10-14-year band (Figure 8B). Since El Niño episodes, characterized by above average precipitation and cooler temperatures in the southwestern United States, are associated with negative SOI phases, the emergence of such anti-phase relationships highlighted the potential of wood anatomical structures to record large-scale climatic variability, confirming at the same time the outcomes of the dendroclimatic analysis performed with local temperature and precipitation data. On the other hand, no clear connections were observed between PDO and LD series (Supplementary Figures S4a,b).

## Discussion

### Intra-annual Wood Cellular Dendroclimatic Relationships

Xylem cellular features of *P. menziesii* were not equally suitable as paleoclimatic proxies. In this study, lumen diameter and CWT differed at both the inter- and intra-annual level in terms of common signal among trees and sensitivity to climate. In contrast to LD, CWT time series lacked common variability (low rbar, EPS, and SNR) and showed high serial autocorrelation (AC1). The high values of AC1 in the chronologies of CWT, particularly in the first sectors (i.e., I and II), might be due to complex dynamics of carbon assimilation and allocation, whose description is beyond the scope of this work, that can be further complicated by external factors, such as disturbance events like drought (Peltier and Ogle, 2020). The low agreement between CWT series observed here and in previous studies (Ziaco et al., 2016), suggests that in drought-prone locations of the Southwest, sub-seasonal internal processes responsible for partitioning of carbohydrates between structural and non-structural sinks (i.e., primary and secondary productivity, storage; Carbone et al., 2013) remains highly variable between trees (Herrera-Ramirez, 2020). On the other hand, the effect of climatic conditions on growth and carbon dynamics in *P. menziesii* (Peltier et al., 2018), as well as in other western United States conifers (Szejner et al., 2018), is known to last for several years. Plant-dependent controls acting on the timing, rate, and duration of cell wall thickening generally reflect a conservative strategy adapted to aridity for maintaining hydraulic integrity (Cuny et al., 2015; Cuny and Rathgeber, 2016). However, under water-limited conditions, *P. menziesii* seems to respond to climatic variability primarily by adjusting lumen size to maintain hydraulic integrity as observed for other conifer species (Carvalho et al., 2015), which makes lumen diameter a valuable paleoclimatic proxy.

Tree-ring sectoring allowed us to enhance the overall quality of dendroclimatic relationships with lumen diameter in *P. menziesii* by targeting the most signal-rich sectors of the ring. This was possible because sampled trees had wide rings and enough cells to make numerous meaningful subdivisions. Intra-annual variability characterized each anatomical parameter at all positions along the ring, especially in the first sectors (i.e., I to V), roughly corresponding to the earlywood. Intra-annual patterns of LD and CWT, as illustrated by 1966–2015 standardized tracheidograms, were characterized by a constant decrease of lumen diameter and gradual thickening of cell walls, without any major, stand-wide intra-annual density fluctuations. LD chronologies showed a consistent inter-annual pattern of synchronous variability among trees in the earlywood, but the common signal gradually decreased approaching the earlywood/latewood transition zone approximately around sector V or VI and disappeared in the later sectors.

Climate-anatomy correlations established for lumen diameter of *P. menziesii* showed the predominant cold-season signal in the first half of the ring, while the contribution of spring climate, although present (see Supplementary Figures S2, S3), was lower. The strongest correlation between daily temperature and lumen diameter found for sectors I to V was with a period ranging from October to January, while it transitioned to a spring–summer signal starting with sector VI (Figure 5). In the southwestern United States, winter temperature may affect snowpack dynamics by favoring snow sublimation, reducing the amount of water penetrating the ground at the time of snow melting (Sheppard et al., 2002). Furthermore, air temperature is tightly related to cloudiness and inversely related to snowy precipitation during the cold season. Hence, previous winter temperature influences the morphogenesis of the first ring cells of each ring (Wang et al., 2002; Martin-Benito et al., 2013; Novak et al., 2013), while summer temperature conditions, and their control on evaporative demand and drought stress in particular, become more important later in the growing season especially around sectors VIII and IX (Cuny and Rathgeber, 2016; Cuny et al., 2019). The similar temporal response to temperature across LD sectors in the first half of the ring suggests that these trees experience fast rates of xylem production in the spring, aimed at maximizing radial growth when evapotranspiration is less demanding, and to avoid the negative effects of spring temperature (Supplementary Figures S2, S3). The previous cold-season precipitation signal persisted, although decreasing in strength, throughout the entire ring. This consistent pattern of correlation across the tree ring confirms the importance of winter precipitation for western conifers (Kerhoulas et al., 2013) and its footprint on wood anatomy even in the core area of the NAM (Ziaco et al., 2018), whereas under non-monsoonal climatic regimes other species tend to show a predominant response to current year precipitation (Ziaco et al., 2016). In line with this, we also found that positive effects of precipitation on lumen diameter persisted until May in sectors I, and within the average of sectors I-V. However, in a short temporal window of 23days (from June 28^th^ to July 20^th^), we observed a temporary inversion in the sign of correlation between precipitation and lumen size (Supplementary Figures S2, S3). However, these two temporal windows showed an overall weaker correlation strength and were not considered useful for further dendroclimatic investigation. In addition to site-specific differences, particularly in seasonal dynamics of moisture availability, it should be noted that discrepancies in the temporal window of climatic responses between our study and previous ones could also be a consequence of parameter selection, i.e., tree-ring partitioning *versus* a whole ring approach, that in this case allowed us to focus on the climatic signal recorded in specific, signal-rich portions of the ring.

Under limiting conditions, the cellular response of tree species to changing moisture conditions is expected to act on lumen size rather than their quantity (Olano et al., 2012). Furthermore, tracheid dimensions are directly dependent on the hydration status of the tree stem during the time of cell formation when water is needed to maintain adequate turgor during the cell enlargement phase (Arzac et al., 2018; Cabon et al., 2020). We observed sensitivity to drought in *P. menziesii*, although the strongest relationship was with previous cold-season climate, which is far from contemporaneous with the timing of the cell enlargement phase, and got weaker with spring conditions. It should be noted that in the mountain ranges of the western United States, winter October–April precipitation falls mainly as snow, contributing to the formation of the snowpack, which in turn determines soil moisture at the beginning of the growing season and the amount of water available for the resumption of cambial processes (Maurer and Bowling, 2014). Hence, snowpack dynamics can affect inter-annual variability of cellular structures and explain a large proportion of the observed variance in earlywood tracheids (Ziaco, 2020). A drought signal in xylem anatomy of *P. menziesii* was demonstrated by the correlation between LD in the earlywood and the SPEI-1 for the months October to January. This correlation is particularly relevant, as the SPEI-1 includes potential evapotranspiration, possibly capturing the effect of water deficit/surplus on tree growth and xylem features (Vicente-Serrano et al., 2010).

### Correlations With Supra-Regional Climatic Patterns

We have shown that annually resolved time series of lumen diameter (LD) hold great potential for linking local temperature and precipitation patterns with large-scale climatic modes in the southwestern United States. This was possible due to the strong climatic signal encoded in cellular structures, and the possibility to enhance the temporal resolution of dendroclimatic correlations using tree-ring partitioning and daily climatic records. Regional correlation fields suggested that the climatic signal encoded in *P. menziesii* tracheids accurately reproduced the same hydroclimatic pattern over the entire American southwest typically shaped by large-scale winter climatic modes (St. George et al., 2010). Weather conditions along western North America are associated with ocean–atmosphere variability over the Pacific Basin, which is responsible for regulating moisture transport at intra-annual to multidecadal time scales (Cook et al., 2004; Potito and MacDonald, 2008; Li et al., 2014). LD in the first half of the tree ring showed positive correlations with winter SST over the central-eastern Pacific, and negative ones over the western and southern Pacific. This correlation pattern reflects the typical conditions leading to El Niño episodes in the United States southwest (Feldl and Roe, 2010). This kind of signal is found in earlywood ring-width chronologies of several conifer species of the southwestern United States, including *P. menziesii* (Stahle et al., 1998), but it has not been observed in time series of wood anatomical parameters.

Teleconnections between different modes of atmospheric circulation over the Pacific Ocean are responsible for a well pronounced NW/SW dipole of precipitation patterns (Brown and Comrie, 2004). Hence, when the PDO is in its constructive (warm) phase, it can strengthen the influence of ENSO and lead to stronger El Niño events, which can amplify the effect on winter precipitation in the southwest (Gershunov and Barnett, 1998). Instrumental records (Mantua et al., 1997) indicate the beginning of a warm phase of the PDO in 1977 with several ENSO events starting right after that period and typically peaking in activity during the northern hemisphere winter. This warm PDO phase leads to wetter than normal conditions over the American southwest, including southern California, Arizona, and New Mexico (Sheppard et al., 2002). By applying a cross-wavelet transform, we found a significant inverse relationship between time series of LD in sector I and I-V with SOI for the period 1980–2005, which corresponds with the last warm PDO phase.

We found no significant LD correlations with climatic parameters that would suggest an influence of the NAM on tracheid size. This was an unexpected result because the NAM is extremely important for relieving summer drought in the southwestern United States, and previous tree-ring network studies indicated a direct NAM effect on the annual variability of *P. menziesii* latewood width (Griffin et al., 2013). The short, intense, and localized nature of NAM thunderstorms mitigates evapotranspiration demands of trees exposed to summer drought, reduces tree water deficit, and by increasing cell turgor potentially promotes the temporal reactivation of cambial activity (Vieira et al., 2014), resulting in cell division and wider latewood bands (Griffin et al., 2013). However, it is likely that on the sloping terrain that dominates the topography of our sites, large volumes of monsoonal rainwater might be lost as runoff instead of recharging soil water storage, limiting the beneficial effects of summer precipitation on tree hydration status (i.e., on cell turgor), and thus reducing its footprint on xylem anatomy, in particular on the radial diameter of newly formed cells, compared to the observed winter-dominant correlation. Furthermore, it should be noted that these sites were selected for their cold-season signal (see Supplementary Figure S1), therefore it is possible that winter, and to a lesser extent early spring precipitation, is generally enough to support growth in this species, making it insensitive to summer precipitation; in fact, decreased sensitivity to monsoonal precipitation after a wet winter has been documented in other western conifers (Ziaco et al., 2018). A recent dendroclimatic reconstruction of seasonal hydroclimate based on earlywood and latewood chronologies has demonstrated that ENSO-driven winter climate is the main driver of precipitation anomalies in this region, also highlighting the weaker signal recorded in latewood chronologies of *Pinus ponderosa* at our study sites (Ziaco et al., 2020). Finally, it should be noted that latewood chronologies of *P. menziesii* from the four corners region show a high correlation with previous earlywood width (Torbenson et al., 2016). In our study, we noticed the absence of first-order autocorrelation for both earlywood and latewood time series of lumen diameter, which may therefore augment precipitation reconstructions based on earlywood and latewood widths without the need to deal with autocorrelation of anatomical parameters (Carrillo et al., 2016).

## Conclusion

Wood anatomical analysis of Douglas-fir growing at high elevations in eastern Arizona showed the dendroclimatic potential of lumen radial diameter. Correlation analyses between intra-annual cell features and climatic conditions suggested that intra-annual cell morphogenesis is driven by climatic conditions during the winter. Cold-season temperature and precipitation were related to radial lumen diameter, while precipitation during the current growing season did not have a significant effect on tracheid dimensions. Snowpack formation during the dormant period and cool-season temperature, affecting the amount of water available at the beginning of the growing season, appeared to be a more relevant limiting factor for intra-annual xylem adjustment than summertime precipitation events and their relieving effect on evapotranspiration demands.

Our findings are species specific and further investigation into the timing and kinetics of cell formation in this species at a similar topographically extreme site would help to refine the strong link observed between lumen size and winter climate and also assist in explaining the missing summer precipitation signal. Spatial field correlations indicated a significant influence of ENSO and, to a lesser extent, PDO patterns on the precipitation regime, particularly when in their “constructive” (warm) phases. By combining high-resolution climatic data and new analytical techniques, we uncovered the potential of cell structural characteristics for reconstructing climatic conditions in mountainous environments with the intention to improve our understanding of teleconnections between climate dynamics in the Pacific region and local hydroclimatic variability in the southwestern United States.

## Data Availability Statement

The raw data supporting the conclusions of this article will be made available by the authors, without undue reservation. All codes for the analyses presented in this document will be made available upon request.

## Author Contributions

DB, IH, FB, and EZ conceived the research design and the sampling procedures. NM and EZ conducted the sample collection. NM developed the ring-width chronologies. DB, HN, and AH performed the wood anatomical sample preparation, measurements, and elaboration of raw data. DB, HN, IH, FB, and EZ performed the dendroclimatic analysis, developed the hypotheses, and wrote the initial draft of the manuscript. All authors contributed to the final version.

## Conflict of Interest

The authors declare that the research was conducted in the absence of any commercial or financial relationships that could be construed as a potential conflict of interest.

## Publisher’s Note

All claims expressed in this article are solely those of the authors and do not necessarily represent those of their affiliated organizations, or those of the publisher, the editors and the reviewers. Any product that may be evaluated in this article, or claim that may be made by its manufacturer, is not guaranteed or endorsed by the publisher.

## Footnotes

^1^ https://climexp.knmi.nl/
