# Supplementary material for: Wood Anatomy of Douglas-Fir in Eastern Arizona and Its Relationship With Pacific Basin Climate
Source: Front Plant Sci. 2021 Sep 1;12:702442. doi: 10.3389/fpls.2021.702442 (PMC8440974; doi:10.3389/fpls.2021.702442)
Supplement: Supplementary file 1 [file Data_Sheet_1.docx]

**Wood anatomy of Douglas-fir in eastern Arizona and its relationship with Pacific Basin climate**

**Balanzategui, D.^1,2,3^, Nordhauß, H.^1^, Heinrich, I.^1,2,3^, Biondi, F.^4^, Miley, N.^4^, Hurley, A. G.^1^, E. Ziaco^1,5*^**

^1^ GFZ German Research Centre for Geosciences, Telegrafenberg, 14473 Potsdam, Germany

^2^ Institute of Geography, Humboldt-University, Unter den Linden 6, 10099 Berlin, Germany

^3^ DAI German Archaeological Institute, Department of Natural Sciences, Im Dol 2-6, 14195 Berlin, Germany

^4^ DendroLab, Department of Natural Resources & Environmental Science, University of Nevada, Reno, NV 89557, USA

^5^ Department of Ecology and Genetics, Plant Ecology and Evolution, University of Uppsala, 752 36 Uppsala, Sweden

**SUPPLEMENTARY MATERIALS**

**Figure S1**

**Figure S2**

**Figure S3**

**Figure S4**

**
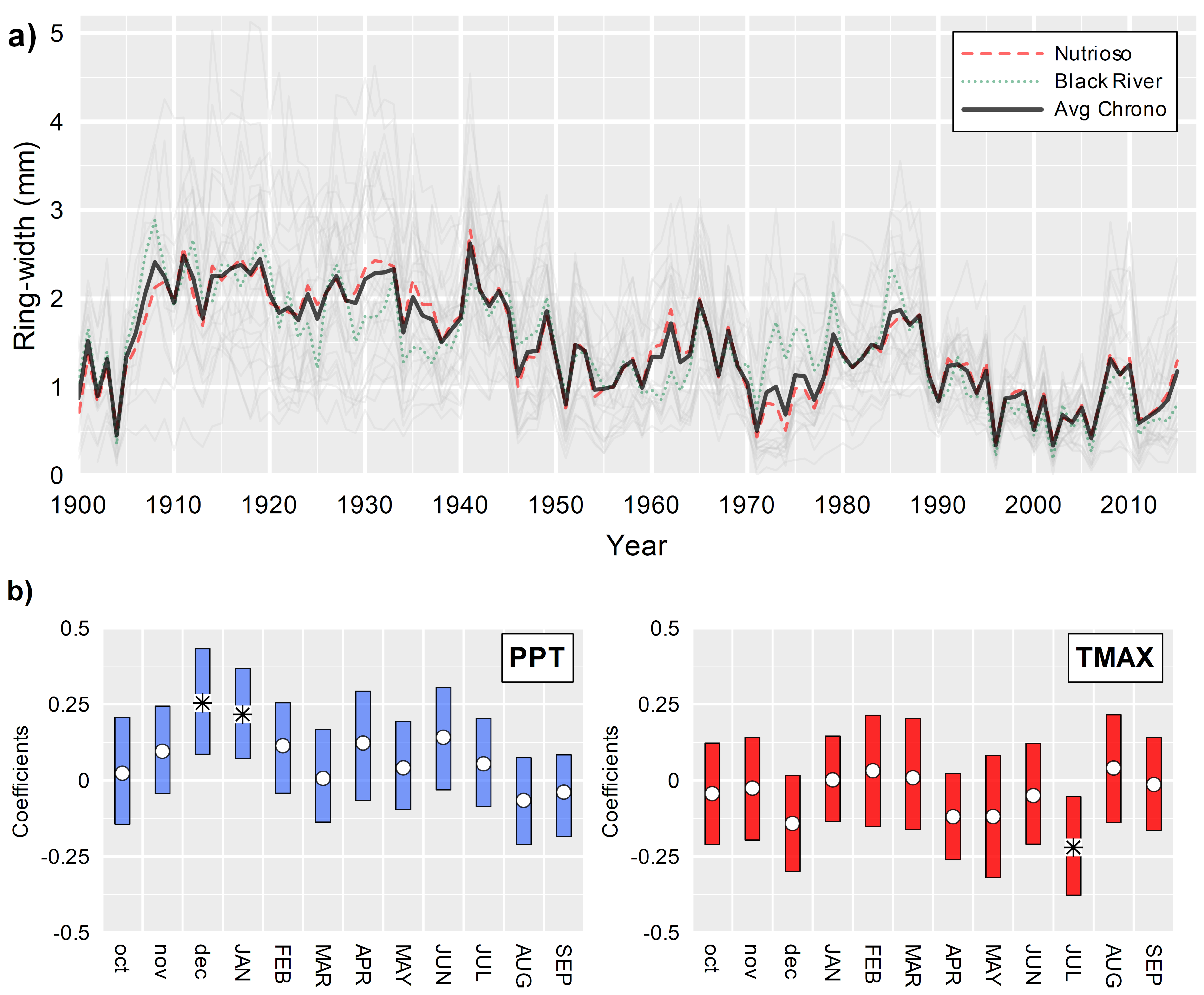
**

**Figure S1** a) Ring-width chronologies for the sites of Nutrioso (red dashed line), Black River (green dotted line), and average of both sites (black bold line); gray lines represent individual ring-width series. b) response function coefficients between residual chronology of *Pseudotsuga menziesii*, monthly precipitation and maximum temperature for the period 1950-2015, calculated for the “water year” (i.e. previous October to current September). Vertical bars represent 95% bootstrapped (n=1000) confidence interval. Significant coefficients are marked with an asterisk. Monthly climatic data were obtained from the PRISM 4-km dataset at the grid point 33.90° N 109.19°W.


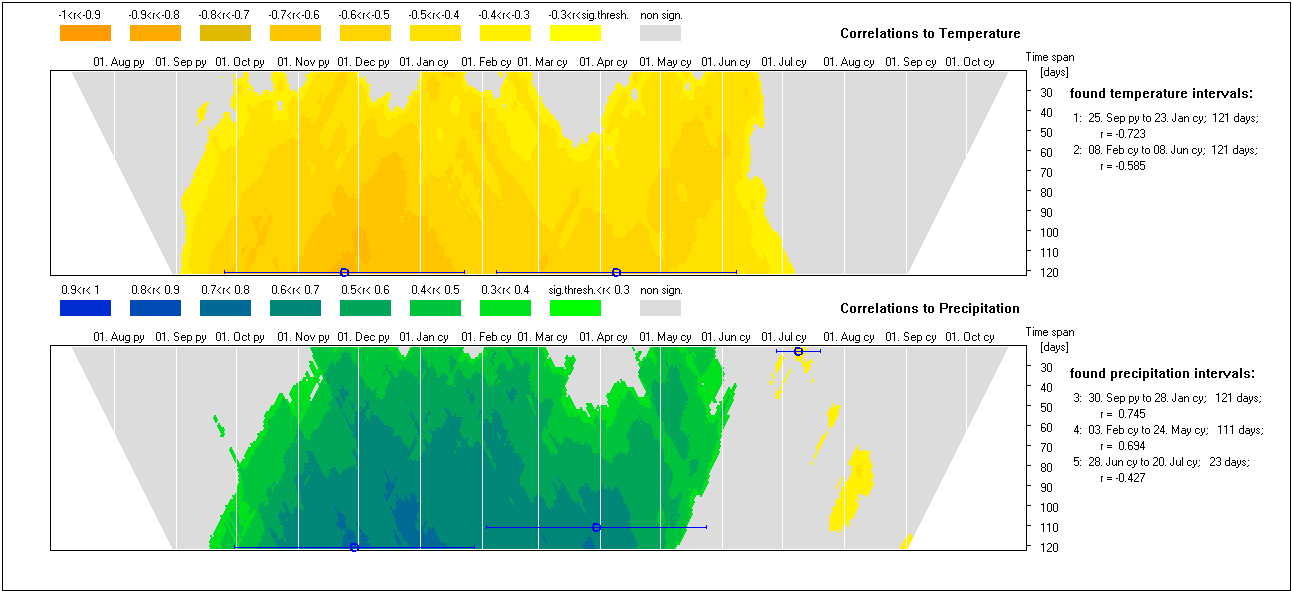


**Figure S2** CLIMTREG analysis output of the dendroclimatic correlations between lumen diameter (Sector I) and daily temperature (top) and precipitation (bottom) for the period 1982-2015. Colors indicate strength and direction of correlations, while horizontal bars indicate the temporal extent of significant climatic windows (*p<0.05*).


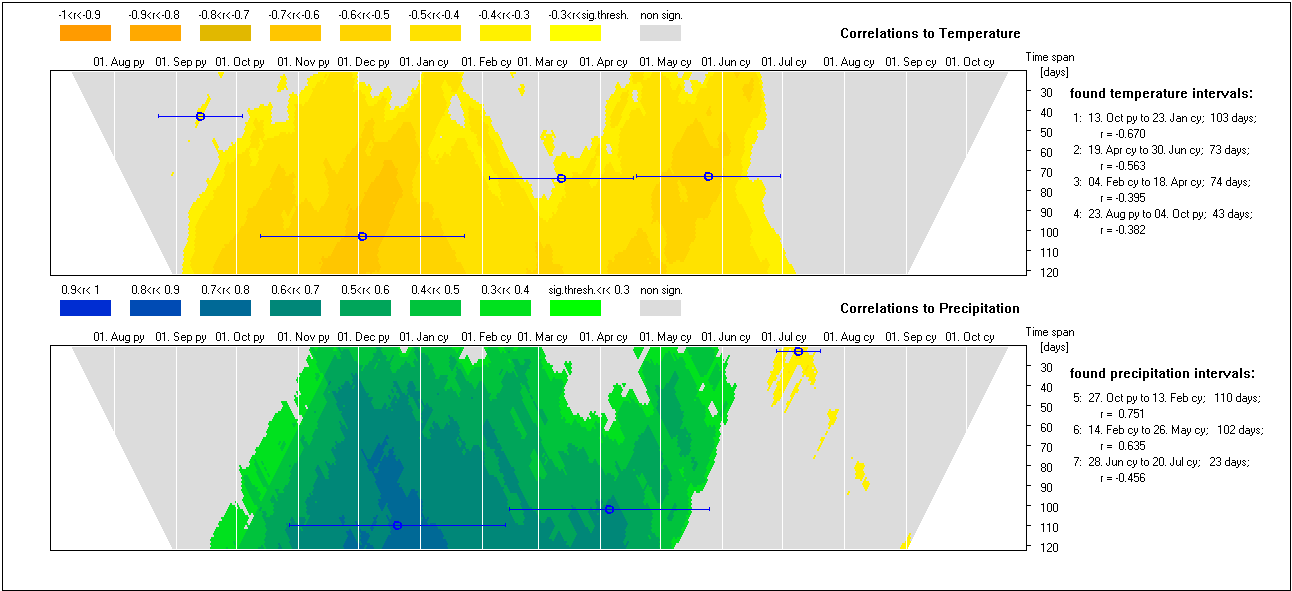


**Figure S3** CLIMTREG analysis output of the dendroclimatic correlations between lumen diameter (Sectors I-V) and daily temperature (top) and precipitation (bottom) for the period 1982-2015. Colors indicate strength and direction of correlations, while horizontal bars indicate the temporal extent of significant climatic windows (*p<0.05*).


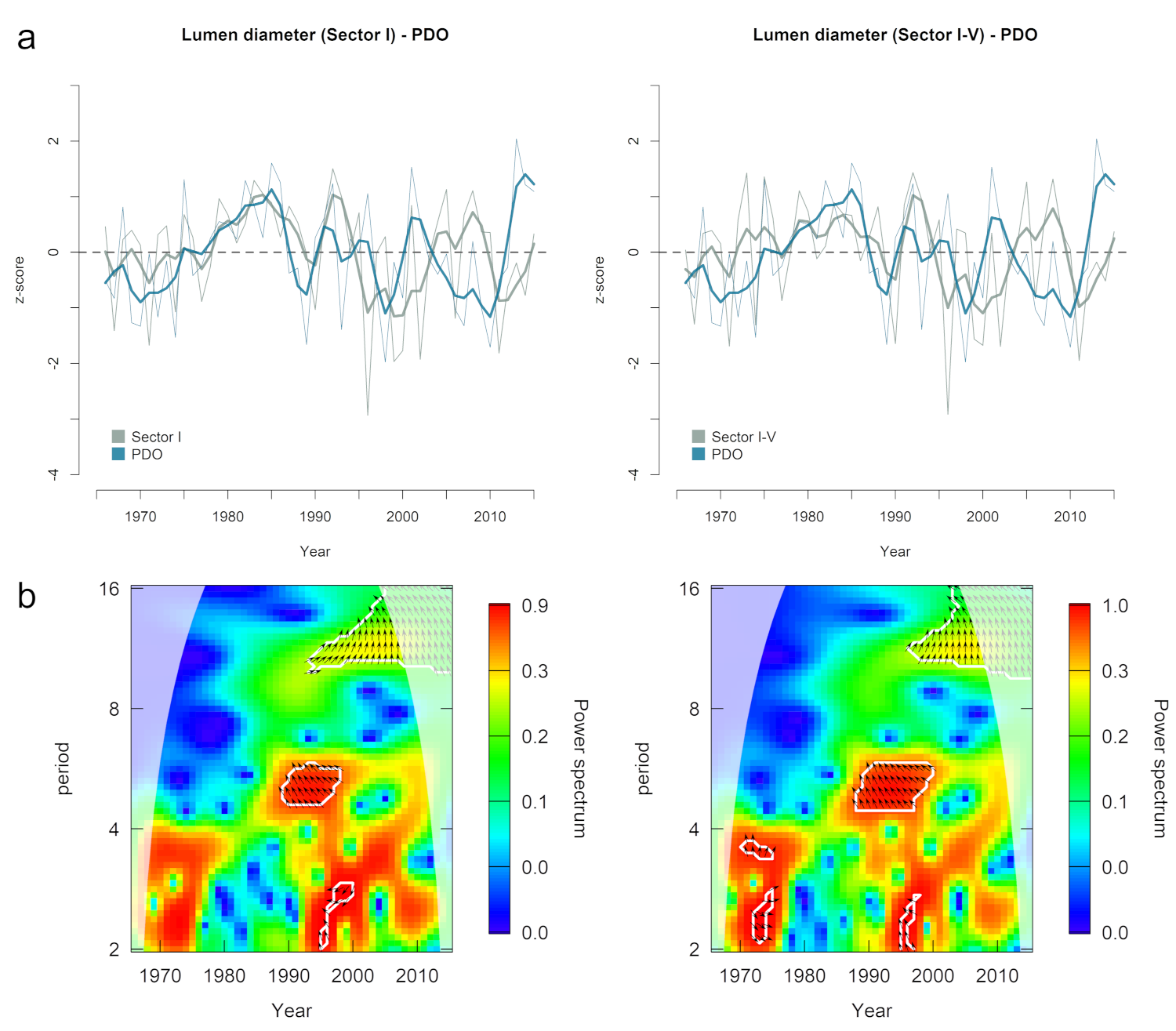


**Figure S4** (a) Normalized time-series of earlywood lumen diameter (Sectors I and I-V) plotted against previous October-current January Pacific Decadal Oscillation (PDO) for the period 1966 to 2015 (bold lines are 5-year cubic smoothing splines). The sign of SOI has been inverted for easier interpretation. (b) Cross wavelet transform of lumen diameter (Sectors I and I-V) and PDO index. White contours represent 95% significance level. The relative phase relationship is shown as arrows (in-phase pointing right, anti-phase pointing left, and PDO leading lumen diameter by 90° pointing down). Results falling outside the cone of influence (white shaded area) might be distorted by edge-effect.
